# Mast Cell-Activated Bone Marrow Mesenchymal Stromal Cells Regulate Proliferation and Lineage Commitment of CD34^+^ Progenitor Cells

**DOI:** 10.3389/fimmu.2013.00461

**Published:** 2013-12-17

**Authors:** Zoulfia Allakhverdi, Michael R. Comeau, Myriam Armant, Rachana Agrawal, Judith A. Woodfolk, Roma Sehmi, Karen J. Howie, Gail M. Gauvreau, Guy Delespesse

**Affiliations:** ^1^Laboratory on Allergy, CRCHUM Notre-Dame Hospital, Montreal, QC, Canada; ^2^Inflammation Research, Amgen Inc., Seattle, WA, USA; ^3^Center for Human Cell Therapy, Immune Disease Institute, Boston, MA, USA; ^4^Department of Medicine, Allergy Division, University of Virginia, Charlottesville, VA, USA; ^5^Firestone Institute for Respiratory Health, McMaster University, Hamilton, ON, Canada; ^6^Asthma Research Group, McMaster University, Hamilton, ON, Canada

**Keywords:** mast cells, bone marrow mesenchymal stromal cells, TSLP, circulating CD34+ progenitor cells, asthma, allergic inflammation, Th2 response

## Abstract

**Background:** Shortly after allergen exposure, the number of bone marrow (BM) and circulating CD34^+^ progenitors increases. We aim to analyze the possible mechanism whereby the allergic reaction stimulates BM to release these effector cells in increased numbers. We hypothesize that mast cells (MCs) may play a predominant role in this process.

**Objective:** To examine the effect of IgE-activated MCs on BM mesenchymal stromal cells which regulate proliferation and differentiation of CD34^+^ progenitors.

**Methods:** Primary MCs were derived from CD34^+^ precursors and activated with IgE/anti-IgE. BM mesenchymal stromal cells were co-cultured with CD34^+^ progenitor cells and stimulated with IL-1/TNF or IgE/anti-IgE-activated MCs in Transwell system.

**Results:** BM mesenchymal stromal cells produce low level of thymic stromal lymphopoietin (TSLP) under steady state conditions, which is markedly increased by stimulation with proinflammatory cytokines IL-1 and TNF or IgE-activated MCs. The latter also triggers bone marrow-derived mesenchymal stromal cells production of G-CSF, and GM-CSF while inhibiting SDF-1. MC-activated mesenchymal stromal cells stimulate CD34^+^ cells to proliferate and to regulate their expression of early allergy-associated genes.

**Conclusion and Clinical Relevance:** This *in vitro* study indicates that IgE-activated MCs trigger BM mesenchymal stromal cells to release TSLP and hematopoietic growth factors and to regulate the proliferation and lineage commitment of CD34^+^ precursor cells. The data predict that the effective inhibition of MCs should impair mobilization and accumulation of allergic effector cells and thereby reduce the severity of allergic diseases.

## Introduction

Although the initial manifestations of a mucosal allergic reaction are localized, recent studies underline a systemic component of allergic diseases and suggest an important role of the bone marrow (BM) in the control of allergic response. Indeed, shortly after allergen exposure BM releases increased numbers of CD34^+^ hematopoietic precursors and differentiated proinflammatory myeloid cells (1–3). CD34^+^ precursors are rapidly recruited to the site of allergen deposition where they display a dual function as both precursors differentiating into eosinophils, basophils, and mast cells (MCs), and proinflammatory effectors releasing high levels of Th2 cytokines/chemokines (4). However, the specific mechanisms underlying airway allergen exposure and communication with the BM leading to augmented numbers of progenitor cells and their increased release remain to be elucidated.

Bone marrow-derived mesenchymal stromal cells (BM-MSCs) are multipotent progenitor cells with immunomodulatory properties [reviewed in Ref. (5)]. Recent studies suggest that BM-MSCs themselves are critical for forming a niche that maintains hematopoietic stem cells (HSCs) and responsible for the production and deposition of the extracellular matrix, the production and concentration of cytokines, and growth factors (6–8). MCs originate from HSCs, which circulate as CD34^+^ precursors until they migrate into tissues where they mature to effector cells. Both CD34^+^ precursors and MCs actively participate in the induction of allergic inflammation by producing high levels of Th2 proinflammatory cytokines in response to inflammatory and epithelial cell-derived cytokines, including thymic stromal lymphopoietin (TSLP) (4, 9). Since MCs are sentinels of the innate immune system and respond very rapidly (within minutes) by releasing different mediators, they are the best candidates for the early signals generated during allergic response that may impact hematopoietic progenitor cell differentiation/mobilization. Here we tested in an *in vitro* model the hypothesis that signals produced by inflamed tissues and local microenvironment at the site of allergic inflammation may have a significant role in determining the communication with the BM stroma and MCs may play important role in this cross-talk.

## Materials and Methods

### CD34^+^ and primary human mast cell cultures

CD34^+^ progenitor cells were positively selected from umbilical cord or adult peripheral blood by double passage through columns (Miltenyi Biotech) leading to cellular preparations containing more than 98% CD34^+^ cells and negative for CD3, CD10, CD14, CD19, CD20, CD40, CD56, CD83, CDw125 (IL-5R), and FcεR1. All samples were collected after informed consent, using protocols approved by the ethic committee at our institution. To obtain MCs, CD34^+^ progenitor cells were cultured in StemPro serum free culture medium (Invitrogen) supplemented with 5 ng/ml of IL-3 and 100 ng/ml of SCF as described elsewhere (10). After 10–12 weeks of culture, >98% of cells were stained for c-kit (BD), FcεRI (e-BioScience), and tryptase (Chemicon). MCs were cultured for 96 h with IgE (1 μg/ml; generous gift of Dr. K. Ishizaka), then extensively washed and crosslinked with anti-IgE (0.5 μg/ml; RayBiotech Inc.) overnight; their supernatants were collected or in some experiments MCs were used in the upper compartment of Transwell system.

Antibodies and recombinant cytokines used included: anti-CD34-APC, anti-CD34-PE, anti-CD117-PE, anti-CD123-PE, anti-CD3-PE, anti-CD14-PE, anti-CD19-FITC, anti-CD20-PE, anti-CD56-PE (all from BD), anti-FcεRI (e-BioScience), anti-IL-5R (generous gift of Dr. Tavernier), polyclonal anti-TSLP (ProScience Inc.), recombinant TNF-α, IL-1β (R&D; 25 and 10 ng/ml, respectively, or 1 ng/ml each when indicated); IL-3, IL-5 (PeproTech; each used at 5 ng/ml). Neutralizing antibody to TSLP (Amgen) and its isotype control antibody were used at 10 μg/ml, as in our previous study (4).

### Primary human bone marrow-derived mesenchymal stromal cells

Human BM-derived mesenchymal stromal cells were established from BM samples (AllCells, Emeryville, CA, USA) by culture in minimum essential medium-α, supplemented with 10% FBS (Hyclone) and 5 ng/ml of basic fibroblast growth factor (FGF; PeproTech). The cells were plated in 24-well plates until confluent, they were then washed with PBS three times to remove the serum components and were co-cultured with CD34^+^ progenitor cells or MCs, as indicated. In the experiment to separate MCs and the stromal cell monolayer, Transwell with a 0.45 μm filter in 24-well plates were used. The supernatants from the various culture conditions were collected and filtered to remove cellular debris. Flow cytometric analysis confirmed that the BM-MSCs expressed CD9, CD10, CD13, CD29, CD44, CD73, CD90, CD105, CD106, and CD166, but not CD14, CD34, or CD45 (all from BioLegend).

### Proliferation assay

CD34^+^ cells were labeled with CFSE and placed in the lower compartment of the Transwell system with or without BM-MSCs; in some experiments, IgE-coated MCs (10^5^ cells/ml) were cultured in the upper compartment of the Transwell system in the presence or absence of anti-IgE. MCs were removed after 6 h of culture. At day 3 of cultures, CD34^+^ cells were gently removed from the BM-MSCs layers (adherent cells) and analyzed for their proliferation by FACS.

### Assessment of cytokine release

Cell-free culture supernatants were analyzed for protein content using commercially available kits, including IL-5, IL-13, G-CSF, GM-CSF, SDF-1, and TSLP (all obtained from R&D).

### Quantitative real-time PCR

RNA was isolated using the RNeasy Mini Kit (QIAGEN). cDNA synthesis was performed using the TaqMan Reverse Transcription kit. The CEBPA gene expression assay ID (from Applied Biosystems, ABI) is Hs00269972_s1. The GATA-2 gene expression assay ID is Hs00231119_m1. Quantitative real-time PCR was performed via TaqMan using ABI gene expression assays on a 7900HT Fast Real-Time PCR System. HPRT was used as a control for cDNA input.

### Immunocytochemistry

Bone marrow-MSCs were cultured on eight-chamber slides until they reached the confluence. The cells were stimulated with or without IL-1/TNF or supernatants of activated MCs (30% v/v), then fixed and stained for TSLP (polyclonal Ab, ProSci Inc.), developed with AEC (Dako) and counterstained with hematoxylin.

### Statistical analysis

Student’s paired and unpaired *t* tests were used when appropriate; *p < 0.05, **p < 0.01, ***p < 0.001.

## Results

### Bone marrow stromal cells produce TSLP

**Figure 1:**
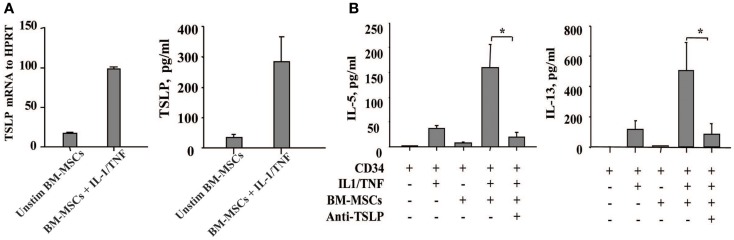
**Bone marrow-derived mesenchymal stromal cells produce TSLP upon stimulation with the proinflammatory cytokines IL-1 and TNF**. **(A)** The expression of TSLP was determined at the mRNA and protein level on the bone marrow-derived mesenchymal stromal cells (BM-MSCs) stimulated with or without IL-1/TNF (10 and 25 ng/ml, respectively); mean ± SEM of five experiments. **(B)** Freshly isolated neonatal CD34^+^ cells were co-cultured overnight with BM-MSCs in the presence or absence of suboptimal concentrations of IL-1/TNF (1 ng/ml of each) and neutralizing antibody to TSLP, as indicated. Isotype control antibody had no biological activity. Supernatants were assessed for IL-5 and IL-13 levels. Mean ± SEM of eight experiments.

Since TSLP is a growth factor released by thymic stromal cells that exerts its effect on proliferation of immature lymphocytes, we have focused on the role of TSLP in the cross-talk of BM-derived mesenchymal stromal cells and CD34^+^ progenitor cells which is likely to take place within the BM stroma. Unexpectedly, we observed that TSLP is expressed both at protein and mRNA levels by BM-MSCs at low levels under steady state conditions and is markedly enhanced in the presence of the canonical proinflammatory cytokines IL-1 and TNF, which mimic inflammatory condition (Figure 1A). BM-MSCs-derived TSLP is biologically active and specific, as it induced IL-5 and IL-13 production by freshly isolated CD34^+^ progenitor cells following overnight cultures with BM-MSCs in the presence of suboptimal concentrations of IL-1/TNF [just sufficient to exert permissive effect on TSLP, as previously published (9)] and it was suppressed by neutralizing mAb to TSLP (Figure 1B). These observations thus suggest that TSLP may be produced under both inflammatory and steady state conditions and could serve as molecular regulator of hematopoiesis and inflammation perhaps via induction of Th2 cytokines by CD34^+^ progenitor cells.

**Figure 2:**
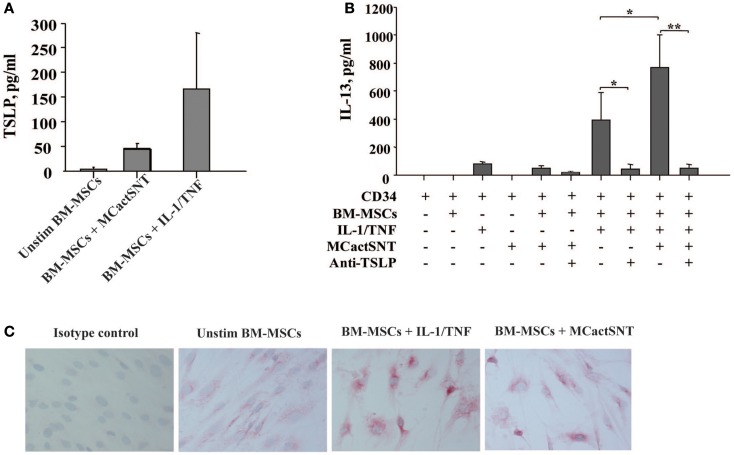
**Activated mast cells induce TSLP production by BM-derived mesenchymal stromal cells**. **(A)** Supernatants of activated (IgE/anti-IgE) mast cells (30% v/v) were added to the BM-MSCs. TSLP expression was analyzed by ELISA. Mean ± SEM of four experiments. **(B)** Freshly isolated neonatal CD34^+^ cells were cultured overnight with BM-MSCs in the presence or absence of IgE/anti-IgE-activated MCs supernatants, suboptimal concentrations of IL-1/TNF (1 ng/ml of each), and neutralizing antibody to TSLP (10 μg/ml), as indicated and supernatants were assessed for IL-13 release. Control isotype antibody had no activity. Mean ± SEM of eight experiments. **(C)** Representative staining of mesenchymal stromal cells cultured under conditions indicated with anti-TSLP polyclonal antibody.

In view of MCs as the first cells to be activated upon exposure to allergen, we next examined whether activated MCs would exert similar effects as IL-1/TNF on the BM. Indeed, the soluble factors released by IgE/anti-IgE-activated MCs induce TSLP production by BM-MSCs as detected by ELISA (Figure 2A). Moreover, the levels of TSLP present in these culture supernatants were sufficient when used together with IL-1/TNF to induce IL-13 production from CD34^+^ cells (Figure 2B). These observations were confirmed by immunocytochemistry showing more intense staining of TSLP protein on BM-MSCs stimulated with IL-1/TNF or supernatants of activated MCs (Figure 2C). Treatment of BM-MSCs with MCs mediators, including LTC_4_, PGD_2_, tryptase, and histamine alone or in combination did not induce TSLP production, which required the combination of all these mediators in the presence of TNF (Table 1).

**Table 1:** **Mast cell mediators’ effect on the release of TSLP by BM-MSCs**.

Conditions	TSLP (pg/ml)
BM-MSCs (−)	18 ± 2.1
TNF	35 ± 5.5
LTC4	N/D
Tryptase	N/D
Histamine	N/D
PGD2	N/D
LTC4/tryptase/histamine	N/D
LTC4/tryptase/histamine + TNF	252 ± 7.8

*N/D, not detected; the results are presented as mean ± SEM*.

### Activated mast cells induce growth factor production by bone marrow mesenchymal stromal cells

**Figure 3:**
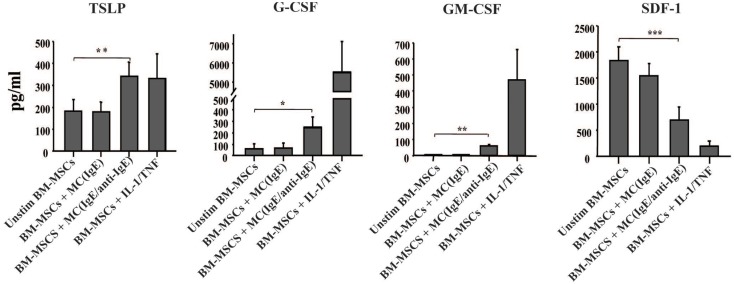
**Activated mast cells induce hematopoietic growth factors production by BM-derived mesenchymal stromal cells**. BM-MSCs were exposed to non-activated and activated MCs in a Transwell system for 6 h, then Transwell membranes with mast cells were removed; the cultures of stromal cells were left for additional 42 h and their supernatants were analyzed for the production of cytokines (pg/ml). Supernatants of BM-MSCs stimulated with IL-1/TNF (10 and 25 ng/ml, respectively) for 48 h were used as a positive control. Mean ± SEM of six to ten experiments.

Since shortly after allergen exposure there is increased proliferation and mobilization of CD34^+^ progenitors from the BM as well as an augmented differentiation of eosinophils, we examined whether activated MCs stimulate BM-MSCs for the increased production of hematopoietic growth factors. In the experiment shown in Figure 3, BM-MSCs were cultured for 6 h with activated MCs in a Transwell system then MCs were removed and BM-MSCs were kept in culture for another 42 h. In addition to TSLP, BM-MSCs released G-CSF and GM-CSF, factors with critical roles in hematopoiesis. Moreover, the production of SDF-1 was significantly reduced in the presence of activated MCs. Attenuation of SDF-1 receptor expression on the BM CD34^+^ cells together with reduction of SDF-1 levels in the BM was shown to regulate the release of progenitors from BM in allergen induced asthma (2, 11). Of note, the supernatants of cultures that were harvested at the point when MCs were removed contained no detectable levels of TSLP, G-CSF, and GM-CSF.

### Bone marrow mesenchymal stromal cells primed with activated mast cells induce proliferation of CD34^+^ progenitors

**Figure 4:**
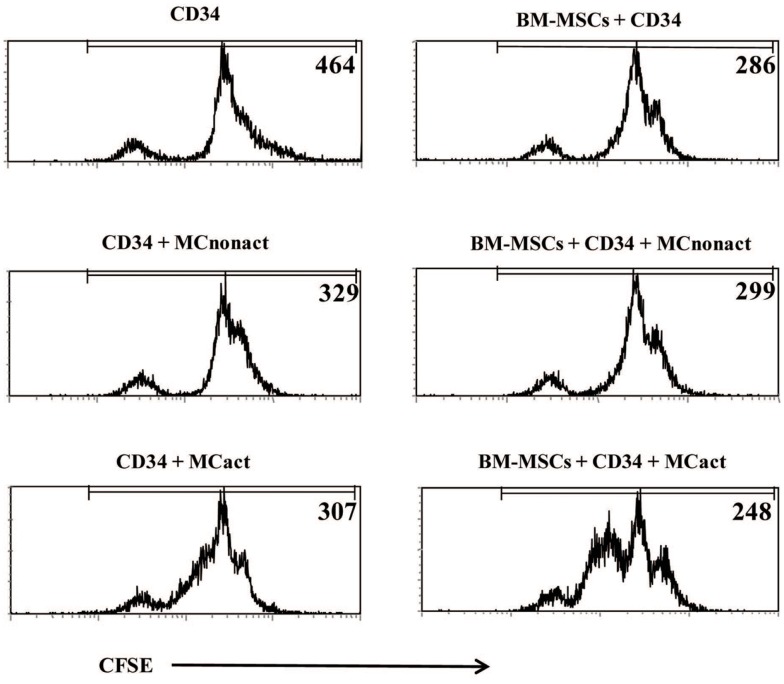
**Bone marrow-derived mesenchymal stromal cells preactivated with mast cells induce proliferation of CD34^+^ progenitor cells and regulate allergy-related genes in CD34^+^ progenitors**. CFSE-labeled freshly isolated CD34^+^ progenitor cells were co-cultured for 3 days with BM-MSCs exposed to activated in a Transwell system mast cells (kept in cultures for 6 h while cross-linking with anti-IgE and then removed) and their proliferation was assessed. Numbers refer to mean fluorescence intensity (MFI) of CFSE-labeled CD34^+^-gated cells. Representative of three experiments.

Because allergen exposure increases proliferation of CD34^+^ progenitors in the BM (12) and at the site of allergen exposure (13), we next analyzed whether activated MCs would have an effect on the proliferation of the progenitors. Indeed, MCs-derived soluble factors enhanced the proliferation of CFSE-labeled CD34^+^ progenitor cells as determined at day 3 of cultures. Non-activated MCs exerted a small but consistent effect on the proliferation of CD34^+^ cells likely due to background levels of activation. BM-MSCs co-cultured with CD34^+^ cells in the lower compartment of the Transwell system also enhanced progenitor proliferation. The proliferation of CD34^+^ cells was markedly increased if BM-MSCs were preactivated by soluble factors released by IgE/anti-IgE-activated MCs in the upper compartment of Transwell, which was removed after 6 h of culture (Figure 4).

### Bone marrow mesenchymal stromal cells primed with activated mast cells regulate allergy-related early genes in CD34^+^ progenitors

**Figure 5:**
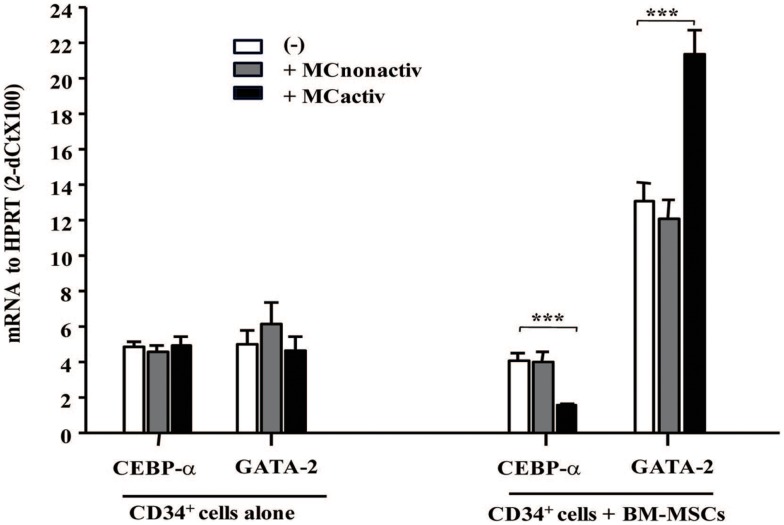
**Bone marrow-derived mesenchymal stromal cells preactivated with mast cells regulate allergy-related genes in CD34^+^ progenitors**. BM-MSCs were exposed to non-activated or activated mast cells in a Transwell system and freshly isolated CD34^+^ cells were added to the cultures for 10 h. Early genes for effector differentiation were analyzed in CD34^+^ cells following culture with BM-MSCs by real-time PCR. Mean ± SEM of four experiments.

To assess whether MC-activated BM-MSCs might prime progenitor cell differentiation into allergic effectors, we have examined the expression of early allergy-related genes in CD34^+^ progenitor cells. After 10 h of co-culture with BM-MSCs pre-stimulated with activated MCs, CD34^+^ progenitor cells demonstrated downregulated CEBP-α and upregulated GATA-2 expression, two genes known to be implicated in the differentiation of progenitors into allergic effectors (14) (Figure 5). Increased expression of GATA-2 in CD34^+^ progenitors gives rise to eosinophil differentiation, whereas a concomitant decrease in CEBP-α and increase in GATA-2 enforces basophil/MC differentiation (15). Collectively, these data suggest that mesenchymal stromal cells stimulated with activated MCs regulate proliferation and lineage commitment of CD34^+^ progenitor cells.

## Discussion

The inflammatory process that occurs following allergen exposure is characterized by the accumulation of immature precursors, eosinophil-committed progenitors, mature eosinophils, MCs, basophils, neutrophils, and CD4^+^ T cells, all of which are of BM origin. The BM of allergic subjects releases increased amounts of CD34^+^ progenitor cells that migrate to the sites of allergic inflammation (2); these cells are readily detectable in the bronchial mucosa and the sputum of asthmatic patients, in nasal polyps and in the lesional skin of atopic dermatitis patients (13, 16). However, the mechanism behind the increased activation of the BM following airway allergen exposure, resulting in both the release of progenitors into the circulation and the production of new effectors is not known. Here we suggest a possible mechanism by which IgE-activated MCs trigger BM mesenchymal stromal cells to release TSLP and hematopoietic growth factors and to regulate the proliferation and lineage commitment of CD34^+^ precursor cells.

In recent years, there has been an increasing appreciation of important contribution of BM-related, hematopoietic mechanisms to allergic diseases. Interesting observations indicated that the BM is able to transfer all allergic manifestations (17, 18) while lung transplantation transfers only asthma (19), thus suggesting that resident BM cells play important role in the regulation of allergic inflammation. Most of the hematopoietic activities in the BM are controlled by the resident mesenchymal stromal cells and their products (20). BM-MSCs have recently been shown to suppress harmful immune responses in patients with steroid-resistant graft-versus-host disease (21), severe systemic lupus erythematosus (22), and cardiac disease (23). The authors of many of these studies concluded that BM-MSCs-driven immunosuppression results from a shift in Th1/Th2 balance (24, 25). Our results showing that cytokines IL-1 and TNF, which mimic inflammatory conditions, and also the products released by MCs following IgE-mediated cross-linking induce production of Th2-inducing cytokine TSLP by BM-MSCs may shed light on these observations. Functional relevance of this observation is demonstrated by increased IL-5 and IL-13 production by CD34^+^ cells and expression of allergy-associated genes by these progenitor cells after interaction with MC-primed BM-MSCs. Importantly, accumulation of eosinophils and basophils in tissues is characteristic of allergic inflammation in rhinitis, nasal polyposis, and asthma (26, 27). These airway tissue inflammatory events may be coincident with relevant changes and fluctuations of circulating and marrow populations of CD34^+^ progenitors following mediators release by BM-MSCs. Indeed, TSLP was suggested to play direct role in the IL-3-independent differentiation of basophils (28). TSLP may also indirectly be implicated in the observed increased eosinophilopoiesis in the presence of IL-1-treated stromal cells (29) by inducing IL-5 release by CD34^+^ progenitor cells. Furthermore, increased proliferation and release of CD34^+^ cells in the presence of MCs-primed BM-MSCs suggest a direct signal between the airway mucosa and BM that is able to increase CD34^+^ production and release at times of increased allergic inflammation. Accordingly, hematopoietic growth factors released by BM-MSCs upon stimulation with activated MCs could explain observed increased proliferation of CD34^+^ cells in asthmatics 24 h after inhaled allergen challenge (30). In addition to their effects on BM-MSCs, MCs products may also act on epithelial cells inducing their release of TSLP (31) and G-CSF (unpublished observations), which may also affect the activity of BM. The finding that the production of SDF-1 by BM-MSCs was significantly reduced in the presence of activated MCs (Figure 3) suggests a possible mechanism for the increased mobilization of CD34^+^ progenitor cells from the BM after allergen challenge, because SDF-1 plays an important role in the homing and retention of progenitor cells to the BM (11, 32). The observation that the MCs mediators could induce TSLP production by BM-MSCs only in the presence of TNF could explain how chronic inflammation increases the sensitization of the airways to the allergen (33, 34). Notably, MCs can release TNF, especially after IgE-mediated activation (35). In keeping with this, TNF was reported to upregulate PAR-2 expression on endothelial cells (36). Although it is tempting to speculate that serine proteases, including tryptase, which is a natural ligand of PAR-2, could be implicated in the induction of TSLP via PAR-2-dependent mechanism, as it was reported for epithelial cells (37), the exact mechanism of TSLP induction by BM-MSCs upon stimulation with activated MCs still remains to be elucidated.

**Figure 6:**
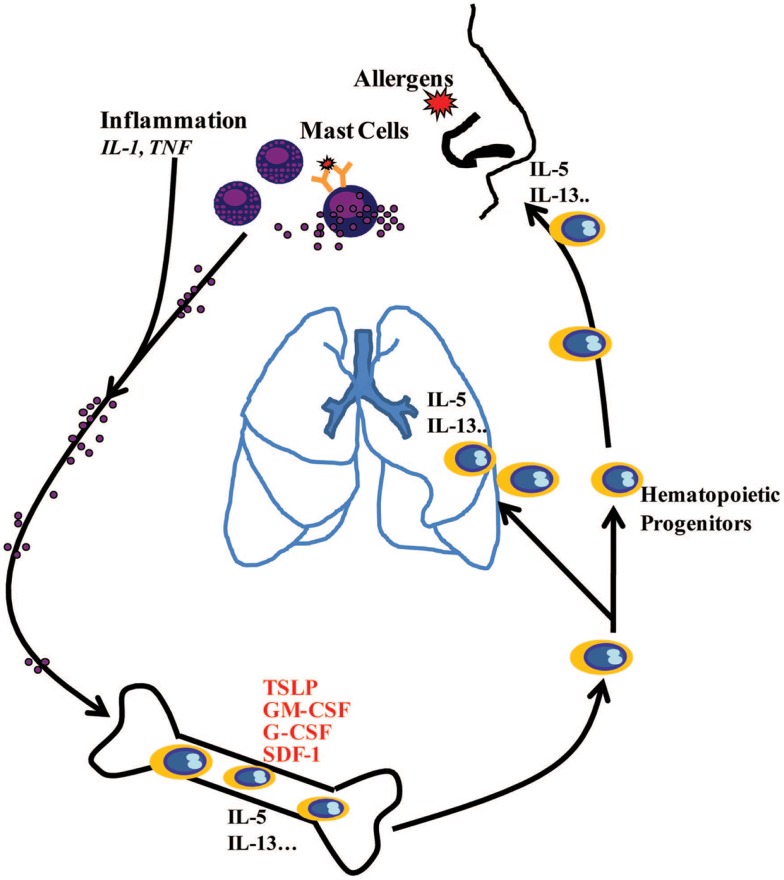
**Schematic diagram showing systemic hematopoietic processes involving active communication between mast cells at the site of allergic inflammation and the bone marrow, highlighting how mast cell-released mediators stimulate the bone marrow to produce hematopoietic growth factors, which in turn regulate proliferation and lineage commitment of CD34^+^ progenitor cells**. CD34^+^ progenitor cells stimulated with MC-primed BM-MSCs may produce IL-5 and IL-13 in the bone marrow and at the sites of allergic inflammation via TSLP-dependent mechanisms.

In conclusion, this study demonstrates that inflammation and IgE-activated MCs stimulate BM-MSCs to release TSLP and other growth factors, which in turn regulate proliferation and lineage commitment of CD34^+^ progenitor cells and also their production of Th2 cytokines. The *in vivo* relevance of these *in vitro* experiments and the obvious question of how MCs-released mediators at the site of allergen exposure reach the BM within minutes remain to be determined. There is an emerging understanding of precisely how this could happen via insoluble particles composed primarily of heparin and cationic proteins, forming extracellular chaperons and protecting these mediators from dilution into interstitial space and degradation (38). Altogether, the present data further underline the important role of MCs in the pathophysiology of allergic diseases by showing that these cells not only display proinflammatory activity at the site of allergen exposure but also induce systemic response involving the BM (Figure 6), as it has been suggested (39).

## Author Contributions

All authors have contributed to the collection of the data. Zoulfia Allakhverdi and Guy Delespesse contributed to the conception and design of the work. Zoulfia Allakhverdi, Michael R. Comeau, Rachana Agrawal, Judith A. Woodfolk, Roma Sehmi, Karen J. Howie, Gail M. Gauvreau, and Guy Delespesse contributed to the data analysis and its interpretation. All authors have approved the submission of the manuscript.

## Conflict of Interest Statement

The authors declare that the research was conducted in the absence of any commercial or financial relationships that could be construed as a potential conflict of interest. Michael R. Comeau is an employee and shareholder of Amgen Inc.

## Abbreviations

BM, bone marrow; BM-MSCs, bone marrow-derived mesenchymal stromal cells; DCs, dendritic cells; MCs, mast cells; SDF-1, stromal derived factor 1; TSLP, thymic stromal lymphopoietin.
